# PB.37: Screen-detected, noncalcified, mammographic lesions with normal or benign ultrasound findings: is stereotactic biopsy necessary?

**DOI:** 10.1186/bcr3537

**Published:** 2013-11-08

**Authors:** D Tzias, S Yusuf, L Wilkinson

**Affiliations:** 1 St George's Hospital and South West London Breast Screening Service, London, UK

## Introduction

Ultrasound has long been used in the symptomatic service, not only to distinguish cystic from solid masses but also to help in the differentiation of benign from malignant lesions. The ability to correlate a benign ultrasound mass with a mammographic mass eliminates the need for further intervention. We evaluate the need for stereotactic biopsy in screen-detected, nonpalpable lesions without calcification, which have either benign or normal sonographic findings.

## Methods

Patients who had stereotactic biopsy for mammographic lesions from January 2011 to January 2013 were retrospectively identified from our screening database. Clinical examination and ultrasound findings, presence of calcification and pathological diagnosis were recorded. Final imaging opinion was also recorded from the pathology request forms.

## Results

Of 4,339 patients recalled for assessment, 1,860 had a biopsy (853 stereotactic and 1,007 ultrasound guided). Stereotactic biopsies were for microcalcification (*n *= 748) and for 105 impalpable, noncalcified densities with normal (*n *= 73) or benign (*n *= 32) ultrasound findings. Malignancy was detected in eight (8%) noncalcified lesions and 169 (23%) microcalcifications (*P *< 0.0002, Fischer exact test). Simple cysts were detected in 28/32 (88%) of cases with benign ultrasound findings. Suspicion of malignancy was mentioned in 38/105 (36%) final imaging opinions. Asymmetry (*n *= 4) and distortion (*n *= 2) were the commonest lesion features associated with a positive biopsy result.

## Conclusion

Stereotactic biopsy for screen-detected mammographic densities with normal or benign ultrasound findings has a low yield of malignancy. Careful analysis of mammographic findings, ultrasound correlation and further multidisciplinary discussion could help reduce unnecessary biopsies.

## References

[B1] Stavros AT Thickman D Rapp CL Dennis MA Parker SH Sisney GA Solid breast nodules: use of sonography to distinguish between benign and malignant lesions Radiology 1995 15 1 123 134 7784555 10.1148/radiology.196.1.7784555

[B2] Lucas-Fehm L Sonographic mammographic correlation Applied Radiology 2005 15 2 20

